# Editorial: Environmental and genomic strategies for conservation and selection in small ruminants

**DOI:** 10.3389/fvets.2024.1396289

**Published:** 2024-04-02

**Authors:** Arianna Manunza, Johanna Ramírez-Díaz, Juan Carlos Rincón Flórez, Tiago Almeida de Oliveira

**Affiliations:** ^1^Institute of Agricultural Biology and Biotechnology, National Research Council (CNR), Milan, Italy; ^2^Department of Animal Science, Universidad Nacional de Colombia, Palmira, Colombia; ^3^Department of Statistics Science, State University of Paraíba, Campina Grande, Paraíba, Brazil

**Keywords:** local breeds, small ruminants, adaptation, selection signatures, conservation, genetic diversity

Small ruminants are one of the first species that have been domesticated ca. 11,000–10,000 years ago (1), and since then their genome has been shaped under several evolutionary pressures. Environmental and anthropogenic factors play a crucial role in changing their genomes and affecting their productive performance: centuries of several breeding strategies led to the development of more specialized breeds often at the expense of indigenous breeds (2). These locally adapted breeds are currently in a critical status of conservation in some places, threatened by constant habitat loss, surviving in small herds and whose existence is seriously damaged (2). The Irish Bilberry goat is a good example of a unique, endangered breed that deserves specific *in situ* conservation efforts (3).

In the last decade, several studies aimed to investigate the adaptation to the local environmental conditions, most of them focused on local or indigenous populations, thus highlighting a probable population-specific selection footprint (4). Detecting regions under selection is a complex task, especially where the natural selection acts leaving detectable fingerprints. This factor is particularly relevant in those breeds that are called “indigenous,” archetypal of the sheep and goat that spread and arrived at every corner of the World. In fact, local breeds are able to survive in a variety of environments, where harsh conditions are the rule, developing specific traits and retaining specific genetic variants (5). Preserving the genetic variability and specific alleles associated with resilience, environmental adaptation and disease resistance is critical for maintaining both genetic health and sustainability of animal genetic resources (AnGR) (6). These locally adapted breeds constitute a genetic reservoir for the future in unexpected climate change scenarios and a cultural heritage. On the other hand, the growing demand to increase the animal production requires research to be focused on maintaining the equilibrium between conservation status and selection of local and transboundary breeds (6). This Research Topic aimed at collecting original studies suitable to improve our understanding of the genetic differentiation of population and breeds, the process of adaptation as well as the mechanisms involved in the expression of important traits and to detect footprints of selection. This information can be transmitted to decision makers to improve animal production without compromising the genetic variability, through specific breeding programs.

In this Research Topic six papers covering the aforementioned aspects, with a balanced contribution from both diversity and selection issues.

Assessing the genetic diversity of populations is essential for developing genetic conservation programs and sustainable breeding strategies. Chessari et al., explored the genetic diversity and population structure of Noticiana sheep, a traditional breed from Sicily never analyzed before using genome-wide variants. Their results demonstrated its genetic distinctiveness, clearly shown by the clustering analysis. The evaluation of the distribution and frequencies of runs of homozygosity revealed a moderately low level of inbreeding and allowed for the identification of several genes and Quantitative trait loci (QTLs) involved in milk and meat production traits. Also, its shared ancestry components with the Comisana breed revealed a common origin. This Research Topic is further enriched with useful information that can help to design and implement conservation strategies *ad hoc* to recover the Noticiana breed and enhance its local products.

Wang et al. explored the genetic variation of three local sheep in the northeastern Tarim Basin (China), and their potential to adapt to a dry, low-rainfall regional environment. Their population structure and selection signature were compared with three introduced sheep breeds, Suffolk, Dorset, and Texel. The genomic differences between native and introduced sheep breeds were assessed by genome-selective scanning, that allowed to detect several genes and their network regulatory mechanisms associated with stress, adaptation to dry and hot environments, disease resistance, growth, and reproduction related traits. These results offer new insights on the processes that regulate the adaptation and drive the selection and once again confirm the value preserving local breeds.

Baazaoui et al. presented a meta-analysis of a worldwide sheep publicly available dataset that is mostly represented by African and Eurasian sheep breeds. The goal of this study was to better investigate the composition and origins of North African sheep. They identified adaptive traits that, while requiring further validation, added new information and clues to improve resilience traits in North African sheep. Resilience to weather variation and heat stress is an important aspect in animal farming (7–9). These insights can find practical applications in the sustainable plans of conservation and management of genetic resources while supporting the local rural economy.

Fernández Álvarez et al. completed a comprehensive investigation about the relationship of casein haplotype variants with morphometry and linear appraisal using a total of 41,323 records from Murciano-Granadina goat. They performed discriminant canonical analysis to study the relationship between the predicted breeding value for 17 zoometric/linear appraisal traits and the individual haplotypes of each component of the casein complex (αS1, β, αS2, and κ-casein). They suggest to routinely test for genotype or haplotype for β casein together with those of αS1 and κ casein, given the importance of these genes in milk production. This allows to identify animals that can potentially transmit desirable traits to their offspring, thus improving the performance in goat production and contributing to the whole dairy industry.

Zheng et al. studied Ganxi goats, a local breed mainly raised in the western part of Jiangxi province (China) with strong adaptability and a high reproductive rate. The authors used transcriptomics to unravel genes involved in the regulation of wound healing via cuproptosis, a new pattern of programmed cell death (10) that has been previously explored but is still unclear. The analyses suggested the lysosome pathway to be a crucial factor for wound healing and cuproptosis, addressing the necessity for additional research in this area.

Finally, a complete review by Aydin et al. on the livestock industry in Turkey concludes this Research Topic. Turkey is a large country in the Middle East, where domestication began. Because of their importance as sheep producers, a comprehensive literature review on the genetic diversity, structure, selection signatures, disease resistance, growth, milk, wool, and reproductive traits, was welcomed in this Research Topic. Based on the most up-to-date census data, breeding and genetic studies, this review provides us with a wide discussion of past genetic studies alongside current challenges on genomic selection and the next steps forward. In this review it is also emphasized the crucial role of indigenous breeds in the Turkish production system and the alarming decline of these breeds due to non-systematic crossbreeding (11).

The ever-growing interest in studying and genetically characterizing indigenous and local small ruminant breeds reveals an encouraging progress toward a more conscious use of the AnGR. These studies frequently emphasize the need to enhance breeding programs with conservation measures to assure the preservation and uniqueness of underutilized breeds. Ranging from conservation to breeding selection strategies, which are often regarded as a paradox, this Research Topic aims to raise awareness, that the balancing and converging of the two practices should instead become a paradigm.

## Author contributions

AM: Writing – original draft, Writing – review & editing. JR-D: Writing – review & editing. JR: Writing – review & editing. TA: Writing – review & editing.
